# Intelligent Fault Diagnosis Framework for Modular Multilevel Converters in HVDC Transmission

**DOI:** 10.3390/s22010362

**Published:** 2022-01-04

**Authors:** Hosameldin O. A. Ahmed, Yuexiao Yu, Qinghua Wang, Mohamed Darwish, Asoke K. Nandi

**Affiliations:** 1 Department of Electronic and Electrical Engineering, Brunel University London, Uxbridge UB8 3PH, UK; hosameldin.ahmed3@brunel.ac.uk (H.O.A.A.); yuyuexiao333@hotmail.com (Y.Y.); wqhhuazi@163.com (Q.W.); Mohamed.darwish@brunel.ac.uk (M.D.); 2 State Grid Sichuan Electric Power Research Institute of China, Chengdu 610094, China; 3 School of Mechatronic Engineering, Xi’an Technological University, Xi’an 710021, China; 4 Visiting Professor, School of Mechanical Engineering, Xi’an Jiaotong University, Xi’an 710049, China

**Keywords:** MMC-HVDC, fault detection, fault classification, principal component analysis (PCA), multiclass support vector machine (SVM), multinomial logistic regression (MLR)

## Abstract

Open circuit failure mode in insulated-gate bipolar transistors (IGBT) is one of the most common faults in modular multilevel converters (MMCs). Several techniques for MMC fault diagnosis based on threshold parameters have been proposed, but very few studies have considered artificial intelligence (AI) techniques. Using thresholds has the difficulty of selecting suitable threshold values for different operating conditions. In addition, very little attention has been paid to the importance of developing fast and accurate techniques for the real-life application of open-circuit failures of IGBT fault diagnosis. To achieve high classification accuracy and reduced computation time, a fault diagnosis framework with a combination of the AC-side three-phase current, and the upper and lower bridges’ currents of the MMCs to automatically classify health conditions of MMCs is proposed. In this framework, the principal component analysis (PCA) is used for feature extraction. Then, two classification algorithms—multiclass support vector machine (SVM) based on error-correcting output codes (ECOC) and multinomial logistic regression (MLR)—are used for classification. The effectiveness of the proposed framework is validated by a two-terminal simulation model of the MMC-high-voltage direct current (HVDC) transmission power system using PSCAD/EMTDC software. The simulation results demonstrate that the proposed framework is highly effective in diagnosing the health conditions of MMCs compared to recently published results.

## 1. Introduction

**Figure 1:**
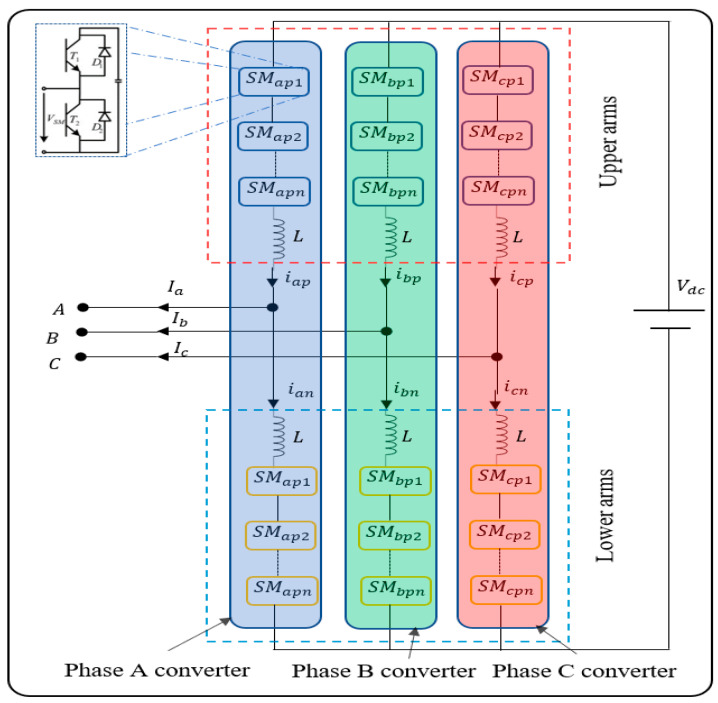
Structure of a three-phase MMC with half-bridge submodules [6].

MMCs in HVDC transmission systems offer high voltage, high efficiency, and high quality of AC voltage [1,2,3]. MMC technology was first introduced by Marquardt [4] in 2001. Recently, it has been implemented by key manufacturers of HVDC equipment and has shown obvious benefits compared with line communicating converters (LCC) based HVDC. The MMCs are essentially constructed by linking several arms to create a three-phase converter. Each arm is created by several series-connected sub-modules built with controllable semiconductor devices such as IGBTs, diodes, and capacitors. In series with each arm, an inductor is added to smooth the current. Based on how the arms are connected, different MMC structures can be developed [5]. Of these, the double-star structure is the most applied topology. Figure 1 illustrates a typical structure of a DC to three-phase AC MMC system with *n* matching submodules (SMs) in each arm of the upper and lower sides of the converters. Each SM is a half-bridge circuit with two IGBTs and one capacitor [6]. Condition classification of MMCs can avoid further substantial failures in the MMC system. Recently, there is an increasing interest in fault detection and localisation of MMC-based HVDC converters. Generally, the fault types can be put into four main types: (1) AC network faults at point of common coupling (PCC); (2) AC faults within the converter station; (3) DC faults such as pole-to-pole or pole-to-ground faults; and (4) converter internal faults such as submodule faults, modulation and control faults, and phase-reactor faults. Yang et al. (2011) have shown that power semiconductor devices were ranked as the key devices for which reliability was of the most concern [7]. A large number of power switching devices used in MMCs to meet the requirement of a high voltage level increases the possibility of the failure occurrence leading to further serious faults [7]. Hence, fault detection and localisation are key issues.

Most voltage-source converters (VSCs) use IGBTs as the power devices because of their high voltage and current ratings, and ability to handle short-circuit currents for periods exceeding 10 μs. MMCs, which utilise VSCs, have common faults in power electronic devices (IGBT, fly-wheel diode), and energy storage capacitors, and cause control failure. Since MMC is made up of hundreds of power electronic devices, IGBT damage is the most common cause of sub-module failure [8]—generally due to short-circuit faults or open-circuit faults [9]. Due to their poor ability to deal with overcurrent, IGBTs must be protected during short-circuit faults, based on short-circuit detection and converter shutdown [10]. Moreover, industrial gate drivers have now typically been integrated with standard short-circuit protections by immediately shutting down the IGBTs when such a fault is detected [11].

Compared to the IGBT short-circuit fault, the IGBT open circuit faults do not essentially cause the system shutdown and can remain undetected for a long time, which may cause overvoltage problems, and may further lead to a secondary fault within the converter or eventually a catastrophic failure of the whole MMC system, consequently resulting in high repair costs [12,13,14,15]. For these reasons, it is essential to develop new fault diagnosis methods that can not only achieve accurate detection and recognition of IGBT open-circuit faults but also reduce the cost of sensing and learning from a large number of measurements. Thus, the main aim of this study is to develop an intelligent fault diagnosis framework for the IGBT open-circuit faults in MMCs.

Conventional multilevel converter fault diagnosis has many techniques to deal with fault detection and localisation [16,17,18,19,20]. For instance, Khomfoi and Tolbert [16] proposed a fault diagnosis and reconfiguration technique for a cascaded H-bridge multilevel inverter drive using principal component analysis (PCA) and neural network (NN). In this method, the genetic algorithm is used to select valuable principal components. Simulation and experimental results showed that their method can detect fault type, fault location, and reconfiguration. Song and Huang [17] proposed a fault-tolerant strategy using H-bridge building block (HBBB) redundancy in cascaded H-bridge multilevel converter (CHMC)-based static synchronous compensator (STATCOM). In this strategy, the fault identification is based on the operator analysis. Simulation and experimental results based on switch-voltage sensed signals appear to validate their method. Sedghi et al. [18] introduced a method for a cascaded H-bridge fault diagnosis using histogram analysis and NN. In this method, the output voltage is used to detect fault type and their locations. Similarly, Jiang et al. [19] proposed a method for a cascaded H-bridge inverter fault diagnosis using discrete Fourier transform (DFT) and NN. In this method, the output voltage is used as a diagnostic signal. Lezana et al. [20] proposed a method for cell fault detection using high-frequency harmonic analysis in a cascaded multicell converter using one voltage measurement per output phase. Furthermore, Yazdani et al. [21] introduced a method for multilevel cascaded converter STATCOM fault detection and mitigation using output phase voltages. The performance of this method is validated using PSCAD/EMDTC simulation. Moreover, Wang et al. [22] presented a strategy for multilevel inverter fault diagnosis based on PCA and multiclass relevance vector machine (mRVM). In this strategy, first, the output voltage is used as a diagnostic signal that is pre-processed using a fast Fourier transform (FFT). Then, the PCA is used to extract fault signatures from the sensed output voltage. With these extracted features, an mRVM model is used to classify fault types.

Even though the efficiency of these methods and many more in fault diagnosis of conventional multilevel converters has been validated in many studies, they cannot be used directly for diagnosing a fault in the MMC system owing to differences in structure and operating principle of MMC and conventional multilevel converters [23]. Hence, other fault diagnosis strategies have been proposed for MMCs. For example, Shao et al. [24] introduced a fault detection method for MMC using a sliding mode observer (SMO) and switching model of a half-bridge. However, the accuracy of measurements may limit its applications. A fault detection and isolation method for open-circuit faults of power semiconductor devices in an MMC based on SMO for the circulating current in and MMC was proposed [25]. Moreover, the detection and location of MMCs’ open-circuit fault using extended state observer (ESO) has been introduced using differences between the theoretical and observed full arm voltages [23]. A fault identification method [26] based on wavelet analysis and NN has been proposed. Jiao et al. [27] presented a method for MMC fault diagnosis using the firefly algorithm optimised support vector machine (FA-SVM), in which FFT-PCA is used to extract features from the voltage signal of a three-phase AC side under the open-circuit fault of IGBT. Kiranyaz et al. [28] proposed a real-time system for early fault detection and identification using adaptive one-dimensional convolutional NN. Furthermore, Li et al. introduced a method for fault diagnosis and location for MMC with the open-circuit fault in SM. In this technique, based on the data character of the basic principle of the MMC in a normal or open-circuit fault condition, a mixed kernel support tensor machine (MKSTM) is offered to dispose of these tensor data [29]. Chen et al. [30] proposed a validation method for the simulation model of a power system integrated with the internet of things that can process high-dimensional simulation data and provide evidence for model error location. The method consists of two main parts. First, a feature extraction method for multivariate time series using factor analysis and modified adaptive Prony method. Second, a validation model based on the similarity evaluation is established. The validation discussed in this paper identifies the model errors and their locations, which can be used to improve the simulation model against the practical/acknowledged system. The method is verified by an application to the MMC-HVDC model in PSASP [30]. Yang et al. proposed two SM failure detection and location techniques, namely, a clustering algorithm (CA)-based technique and a calculated capacitance (CC)-based technique. In the proposed CA-based method, the K-means clustering algorithm is employed to detect and locate the faulty SMs with open-switch failures through identifying the pattern of 2-D trajectories of the SM characteristic variables. The proposed CC-based method is based on the calculation and comparison of a physical component parameter—namely, the nominal SM capacitance—and is capable of failure detection, location, and classification within one stage [31].

Recently, Huai et al. [32] introduced a single-ended fault location technique with variational mode decomposition (VMD) method and novel parameter optimization scheme using traveling wave (TW) that could be used as an alternative backup plan for communication-based fault location methods. In this method, the VMD is utilised to extract fault features for fault location estimation, which is further improved by using singular entropy-based parameter optimization. The continuous TW arrivals can be reflected via the most singular IMF under the optimized VMD [32]. Han et al. [33] presented a fault diagnosis technique based on short-time wavelet entropy integrating the LSTM and the SVM. In this technique, the short-time wavelet entropy is used to extract the fault information, then the LSTM process theses information, and finally the output of the LSTM is used as input of the SVM to obtain the fault diagnosis result [33]. Xing et al. [34] proposed a machine learning-based detection and location method for open-circuit fault. In this method, sliding window and feature extraction were used to construct the dataset, which was then used to train a model based on multivariate Gaussian distribution for anomaly detection [34]. Ye et al. [35] introduced a technique for fault location of MMC-HVDC using wavelet transform and deep belief network (DBN). In this technique, first, the wavelet transform is used to decompose the original single pole ground fault voltage waveform, and then the computed high frequency and low-frequency components were used to train different DBN models for fault location estimating [35]. Shen et al. [36] presented a fault diagnosis method for the IGBT open-circuit faults of different MMC SMs have similar characteristics using weighted-amplitude permutation entropy (WAPE), DS evidence fusion theory, and the LSTM techniques [36]. Ke et al. [37] proposed a fault diagnosis technique for MMC based on the synchro-squeezing transform (SST) and genetic algorithm optimized deep convolution neural network (GA-DCNN). In this technique, first, the time-frequency representations (TFRs) of the raw signals which are synthesized by AC, and the inner circulating current of the MMC are calculated with SST. Then, DCNN is employed to learn the underlying features from the TFRs. The genetic algorithm is used to optimize the key hyperparameters of the DCNN while batch normalization, dropout, and data augmentation technologies are investigated to prevent the DCNN model from overfitting and improve the performance of the model [37]. More recently, Guo et al. proposed a fault diagnosis framework for MMC using temporal convolutional network (TCN) integrating adaptive chirp mode decomposition (ACMD) and silhouette coefficient (SC). In this technique, first, the ACMD is used to extract and reconstruct signal components from the original signal. Then, SC is used to characterize the importance of each component. Finally, the TCN model was employed to automatically extract features from the signal components and provide the classification results [38].

Various HVDC open-circuit fault diagnosis-based studies rely on measurements collected from voltage sensors [11,39,40] or both voltage and current sensors [26,41,42,43] within the IGBT modules. In [39], a sensor-less current measurement technique is used. It is mainly based on measuring the instantaneous sub-modules capacitor voltages with the aim to suppress the circulating second harmonic current rather than detecting an open-circuit fault. In [40], a reduced number of voltage measuring techniques are used, but an individual voltage sensor is still needed for each IGBT module. In [11], a method based on the theoretical assumption that all measurements are available and are fed to a MATLAB/Simulink model is used. Reference [41] is based on fault diagnosis techniques using both voltage and current sensors. Reference [26] can accurately locate the bridge arm of the faulty submodule but cannot locate the specific sub-module. Reference [42] has the limitation of failing to identify how many IGBTs failed when multiple faults occur simultaneously. Reference [43] uses excessive current measurements in each arm and the voltage measurements across each submodule. Certainly, there will be huge advantages if a minimum number of only current sensors are used to detect faults with high accuracy and reduced computation time. This is covered in our paper.

Table 1 records different techniques used for IGBT open circuit fault detection and localisation [24,42,44,45], based on threshold parameters. These have difficulties in setting correct threshold values for different operating conditions. In contrast, AI-based techniques can automatically develop a model and improve the accuracy of fault diagnosis. Here, the fault diagnosis of the IGBT open circuit in SMs is studied to overcome the aforementioned problems and achieve high fault classification accuracy with fewer sensors and reduced computational time.

We propose an intelligent diagnosis framework for MMCs faults using machine learning-based technique and combined information of current sensors to automatically recognise the open-circuit failures of IGBT in MMCs. The contributions of this paper are as follows:

- The proposed intelligent fault diagnosis framework utilises only current sensor data for fault diagnosis. This study achieves high accuracy of fault diagnosis using only current sensors and AI-based techniques.
- We combine the measured current data of the AC-side three-phase current and of the upper bridge and lower bridge of each three phases to form a vector of features that represent the current health condition of MMCs.
- Our proposed framework reduces measured current data using PCA that linearly maps the current data into a lower-dimensional space of principal components.
- For fault classification, multiclass SVM based on error-correcting output codes (ECOC) and multinomial logistic regression (MLR) algorithms are used with the learned feature vector to achieve improved classification accuracy and reduced computation time.
- Compared to recently published results that are based on machine learning techniques, our proposed method is faster and yet achieves competitive, if not better, classification accuracies of open-circuit failures of IGBT fault diagnosis in MMC-HVDC transmission.
- The high reduction in the computational time comes from two elements of our proposed method: (i) using a minimum number of only current sensors; (ii) using the PCA method to select fewer features that can be used for training the classification algorithm—i.e., SVM and MLRC algorithms—and for classifying MMC-HVDC health conditions using the trained classification models.
- Being able to obtain high classification accuracy while highly reducing the computational time, our proposed method can be used in real implementations of MMC-HVDC fault diagnosis systems.

The use of only current signals of the AC-side three-phase current and the upper bridge and lower bridge of each three phases for open-circuit fault detection and classification of MMCs has been investigated in our recently published papers [6,46]. We have conducted a series of investigations using the same dataset and deep learning methods to identify the fault type. In [6], we discussed autoencoder-based deep neural network (AE-DNN) and convolutional neural networks (CNNs) methods. In [46], we investigated long short-term memory (LSTM) neural network methods. In this paper, our proposed framework reduces measured current data using PCA that linearly maps the time series data into a lower-dimensional space of principal components that retain most of the variance of the original measurements of the current signals. Then, for fault classification, ECOC-based SVM and MLR algorithms are used with the learned feature vector to achieve improved classification accuracy and reduced computation time. The experimental results demonstrate that our proposed framework with MLR is much faster than the CNN, AE-DNN, LSTM, and BiLSTM that were studied in [6,46]. For example, the results illustrate that our proposed framework with MLR requires only 1% of the time of the CNN, 0.27% of the time of the AE-DNN, 0.6% of the time of the LSTM, and 0.3% of the time of the BiLSTM, and yet our classification results are better.

The remainder of this paper is organised as follows. Section 2 describes the proposed framework including the model of the MMC-HVDC used to generate the data in PSCAD/EMTDC, and the framework for fault detection and classification of the IGBT open circuit fault in SMs of MMC-HVDC systems. Section 3 is devoted to descriptions of the experimental study used to validate the proposed framework. Section 4 presents comparison results. Finally, Section 5 offers some conclusions.

## 2. Proposed Framework

### 2.1. Data Modeling

**Figure 2:**
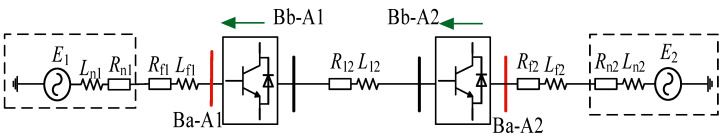
Structure of the HVDC.

The data for this study was simulated from a two-terminal model of the MMC-HVDC transmission power system using PSCAD/EMTDC [47]. It solves the differential equations of the entire power system and its controls. Figure 1 shows that each phase of the three-phase MMC consists of two arms (upper and lower) that are connected to two inductors *L*. Each arm contains a series of SMs, and each SM involves two IGBTs (i.e., $T_{1}$ and $T_{2}$), two diodes *D*, and a DC storage capacitor. In our model (Table 2), different locations of IGBT break-circuit fault are set manually for each bridge (namely A-phase lower SMs, A-phase upper SMs, B-phase lower SMs, B-phase upper SMs, C-phase lower SMs, and C-phase upper SMs). There were 100 cases of IGBT break-circuit fault that happened at different IGBTs of the six bridges at different times. The power system is depicted in Figure 2. The type of SMs is half-bridge and the direction of the flow is shown as the green arrow. Ba-A1 and Ba-A2 are two AC current bus bars. Bb-A1 and Bb-A2 are two DC current bus bars. E_1_ is an equivalent voltage source for an AC network. E_2_ is a wind farm.

The whole time period used is 0.1 s while the time for the IGBT open circuit fault duration is varied from 0.03 s to 0.07 s. The simulation time step is 2 μs and the sampling frequency is 20 kHz. The data recorded for fault diagnosis are AC-side three-phase current (*I*_a_, *I*_b_, *I*_c_) and three-phase circulation current (*I*_diffa_, *I*_diffb_, *I*_diffc_). The circulation current and bridge current can be represented mathematically using the following equations, where *k* stands for the *a*, *b*, and *c* phase, while *p* and *n* separately stand for the upper and lower arms of the MMC. The symbols *i_kp_* and *i_kn_* are respectively the currents of the upper bridge and lower bridge of each three phases.

(1) $$\{\begin{array}{c} i_{k_{p}}=i_{d_{iffk}}+\frac{1}{2}\;i_{k}\\ i_{k_{n}}=i_{d_{iffk}}-\frac{1}{2}\;i_{k} \end{array}$$

(2) $$i_{d_{iffk}}=\frac{1}{2}\;(i_{k_{p}}\;+\;i_{k_{n}})$$

**Figure 3:**
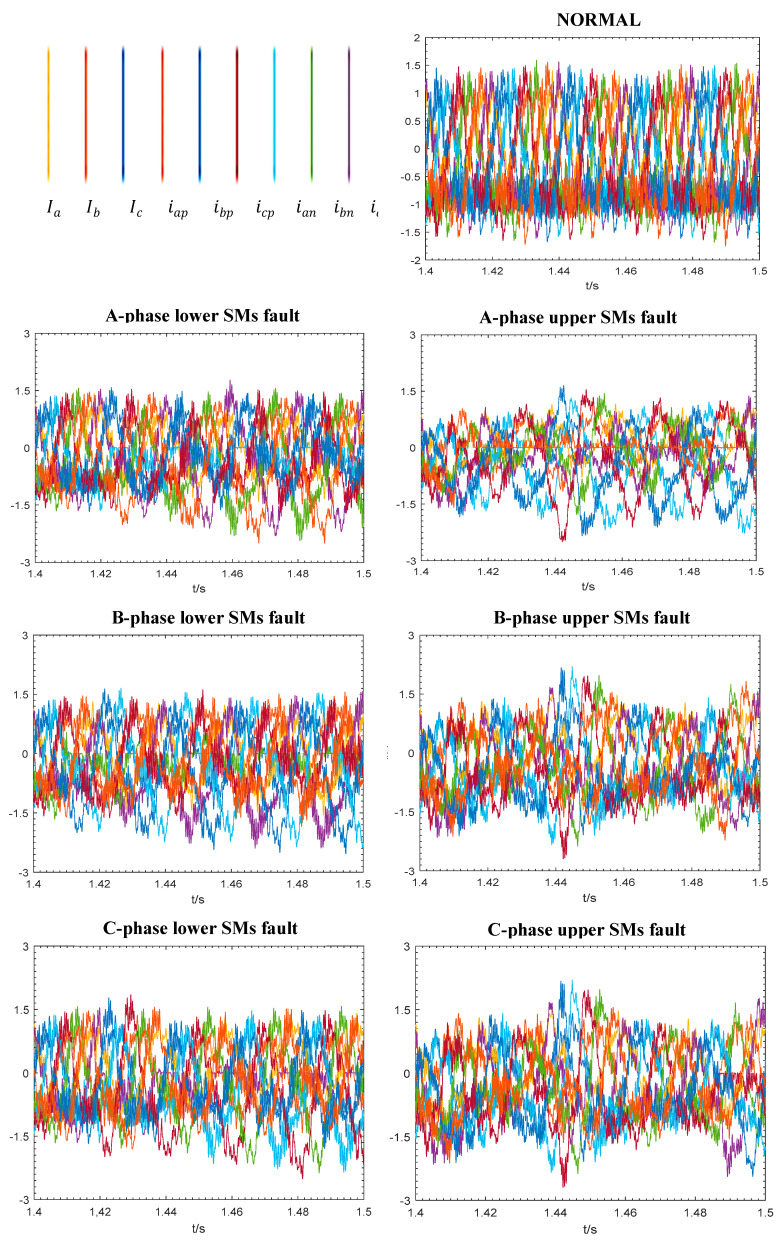
Typical time series plots for seven different conditions as shown in Table 3.

Since the values of *i_ap_*, *i_bp_*, *i_cp_, i_an_*, *i_bn_*, and *i_cn_* can be directly measured, we recorded them, instead of *I*_diffa_, *I*_diffb_, and *I*_diffc_. Consequently, we recorded nine parameters, i.e., *I*_a_, *I*_b_, *I*_c_, *i_ap_*, *i_bp_*, *i_cp_*, *i_an_*, *i_bn_*, and *i_cn_*, (see Figure 1). Figure 3 depicts some typical time series plots for seven different health conditions of the MMC as shown in Table 3, based on the values of the nine parameters described above during the seven states of the wind farm side MMC. Depending on the fault conditions, the defects modulate the recorded signals with their own patterns. The plots give an example of every kind of state, in which six types of faults happened at different IGBTs at different times.

### 2.2. Description of the Proposed Framework

**Figure 4:**
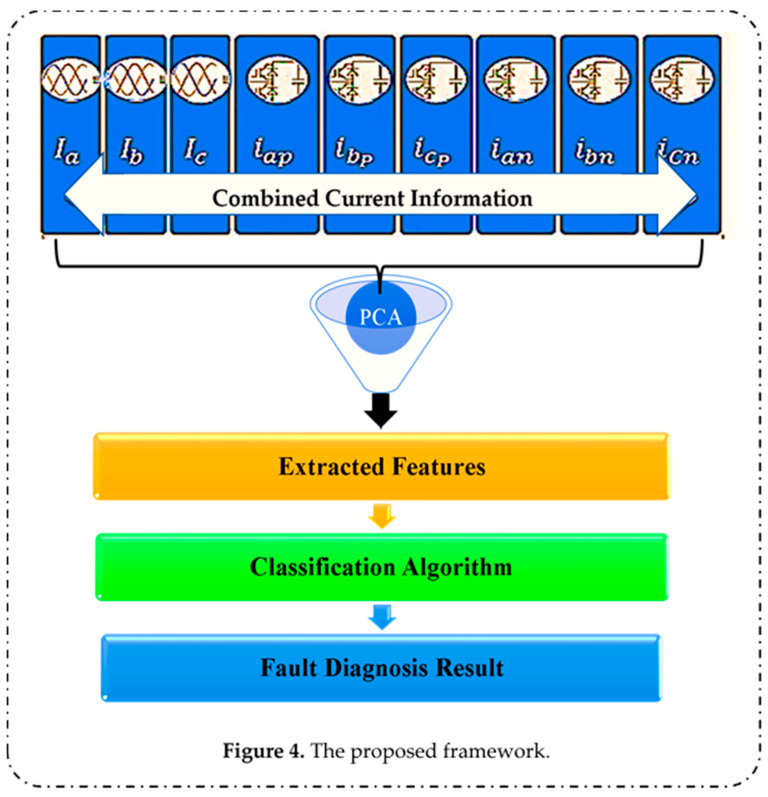
The proposed framework.

Our proposed framework for fault detection and classification of the IGBT open circuit fault in SMs of MMC-HVDC systems is presented here. As illustrated in Figure 4, first, nine parameters—i.e., *I*_a_, *I*_b_, *I*_c_, *i_ap_*, *i_bp_*, *i_cp_*, *i_an_*, *i_bn_*, and *i_cn_*—are recorded. Then, we combine the measured current data of the AC-side three-phase current and three-phase circulation currents to form a vector of features. However, they may not be the best features from a classification point of view. Moreover, in real operating conditions, the size of the acquired and combined data represents a large amount of data to be processed for monitoring of SM health conditions. Therefore, a technique to extract a set of features that achieves superior fault detection and diagnosis, and consequently reduces the computational cost, is needed. Our framework employed PCA [48], often used for feature extraction and dimensionality reduction, to extract principal components from the measured current data.

The PCA [48] is used to form a low-dimensional feature vector from the high-dimensional combined current data by ignoring the least significant of these components from the PCA. The process of PCA using eigenvector decomposition includes the following steps: First, calculate the mean vector of the combined measured data. Then, compute the covariance matrix of the combined measured data. Finally, obtain the eigenvalues and eigenvectors of the covariance matrix. Suppose that the combined measured dataset $X=\left[x_{1},x_{2}\ldots \;x_{L}\right]$ has *L* observations and *N*-dimensional space. PCA transforms *X* to $\hat{X}=\left[\;{\hat{x}}_{1},\;{\hat{x}}_{2},\;\ldots ,\;{\hat{x}}_{L}\right]$ in a new m-dimensional space. The aim is to reduce the dimensionality from  N to m (m≪N) of measurements. To calculate the transformation matrix  W, PCA utilises eigenvalues and eigenvectors as

(3) $$\lambda \vec{V}=C_{X}\vec{V}$$

Here, λ is an eigenvalue, $\vec{V}$ is an eigenvector, and $C_{X}$ is the related covariance matrix of the combined data *X*, which can be computed by applying the following Equation (4).

(4) $$C_{X}=\frac{1}{L}\;\overset{L}{\underset{i=1}{\sum }}(x_{i}-\bar{x}){\left(x_{i}-\bar{x}\right)}^{T}$$

where $x_{i}$ is observation (*i*) of the combined measured data and $\bar{x}$ is the mean of the observations, which can be calculated using the equation,

(5) $$\bar{x}=\frac{1}{L}\;\overset{L}{\underset{i=1}{\sum }}x_{i}$$

To reduce the dimensionality of our combined data X by means of PCA using eigenvector, one ignores the least important components from the principal components, i.e., columns of  V, as

(6) $$\hat{X}=W_{1}^{T}x\;$$

Here $\;\hat{X}\in R^{L\;x\;m}$ is the obtained low-dimensional data matrix of our combined data x, and $W_{1}$ is the projection matrix.

Finally, with these extracted features, SVM-based ECOC and MLR algorithms are used for classification.

#### 2.2.1. SVM-Based ECOC

SVM is a supervised learning algorithm that was first proposed for binary classifications [49]. The basic idea of SVM is that it can find the best hyperplane(s) to separate data from two different classes in such a way that the distance between the two classes, which is called the margin, is maximised. The basic SVM classifier is constructed from a simple linear maximum margin classifier. Briefly, we present the simplified SVM classifier as follows:

With a set of examples of the obtained low-dimensional data of our combined data, i.e., the selected features from PCA, ($\hat{X}\in R^{L\;x\;m}$) along with their associated class $C\in R^{L\;x\;1}$ such that,

(7) $$\hat{X}=\left(x_{1},\;c_{1}\right),\;\left(x_{2},\;c_{2}\right),\;\ldots ,\;\left(x_{L},c_{L}\right)$$

(8) $$=\left(x_{i},\;c_{i}\right),\;\;\;\;\;\;\;\;\;\;\;\;\;\;,\;\forall i=1,2,\;.\;.\;.,\;L$$

Here $x_{i}\in R^{m}$ is an *m*-dimensional input vector that represents the observation (i) of the low-dimensional matrix and $c_{i}$ is the associated class label for every i=1,2, . . ., L. Assume $c_{i}\in \left\{+1,-1\right\}$ for two possible classes ‘NORMAL’ and ‘FAULTY’ conditions, which is a binary classification problem. Binary classification aims to define a function f(x) that can successfully predict the class label $c_{i}$ for an input vector $x_{i}$ by defining a hyperplane, which is also called a boundary, that can separate the examples with the NORMAL class from the examples with the FAULTY class such that

(9) $$\;\;\;\;\;\;f\left(x\right)=sgn\left(w^{T}x+b\right)$$

Here  sgn is the sign of $\left(w^{T}x+b\right)$, x is the input vector, w is an *m*-dimensional vector that defines the boundary, and b is a scalar. To find the best boundary the following three hypothesizes are defined,

(10) $$H_{0}=w^{T}x+b=0$$

(11) $$\;H_{1}=w^{T}x+b=-1$$

(12) $$\;H_{2}=w^{T}x+b=+1$$

**Figure 5:**
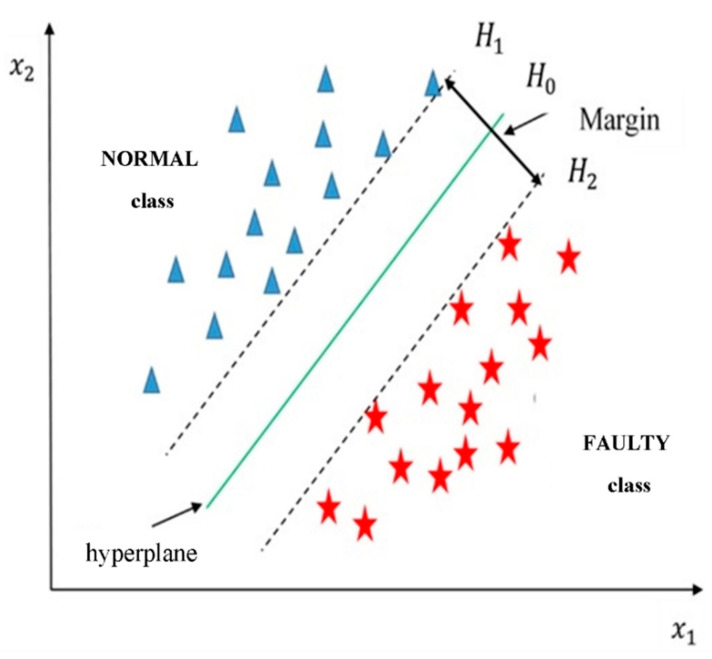
Example of a linear classifier for a two-class problem (40).

As shown in Figure 5, the distance between $H_{1}$ and $H_{2}$ is known as a margin. The best classifier would be the one with the largest margin—i.e., the larger the margin is, the better is the classifier.

The model described above is meant to be used for linearly separable data. Non-linear classifications can be performed by employing various kernel functions, e.g., radial basis function (RBF), polynomial function (PF), and sigmoid function (SF) [50]. In this study, our classification problem is a multi-class classification problem with seven health condition labels. Several binary SVM classifiers can together deal with the multiclass classification problems using various techniques such as one-against-all, one-against-one, and direct acyclic graph (DAG). In this research, we employed the “fitcecoc” function [51] on the selected features from PCA. The “fitcecoc” function uses [c(c−1)/2] binary SVM models, using a one-versus-one coding design, where c is the number of unique class labels. This will return a fully trained error-correcting output code (ECOC) multiclass model that is cross-validated using 10-fold cross-validation.

#### 2.2.2. MLR

Regression analysis can be utilised for predictive modelling by defining the relationship between a dependent variable and one or more independent descriptive variables. There are several types of regression analysis techniques including linear regression, polynomial regression, logistic regression, etc. Logistic regression (LR) is one of the most widely used techniques in binary classification problems. Briefly, we describe the LR as follows:

Let a set of examples of the obtained low-dimensional data of our combined data, i.e., the selected features from PCA, ($\hat{X}\in R^{L\;x\;m}$) along with their associated class $C\in R^{L\;x\;1}$ as described in Equations (7) and (8) above. The LR is a probabilistic discriminative method that learns P(c|x) directly from the training where $c_{i}\in \left\{0,\;1\right\}$ such that

(13) $$P\left(c=1|x\right)=h_{1}\left(x\right)=g\left(-\theta^{T}h\right)=1/\left(1+e^{-\theta^{T}q}\right)$$

Here *c* = 1 is the class, x is the input observation of the obtained low-dimensional data, and $g\left(-\theta^{T}h\right)$ is the logistic function. As ∑P(C)=1 then P(c=0|x) can be calculated as

(14) $$P\left(c=0|x\right)=h_{0}\left(x\right)=1-\;P\left(c=1|x\right)=1-\left(1/\left(1+e^{-\theta^{T}x}\right)\right)$$

The likelihood of the parameters of *L* examples of the data can be calculated as

(15) $$\ell \left(\theta \right)=\overset{L}{\underset{i=1}{\prod }}({\left(g\left(\theta^{T}x^{i}\right)\right)}^{c\left(i\right)}{\left(1-g\left(\theta^{T}x^{i}\right)\right)}^{1-c\left(i\right)}\;$$

Here $\theta =\left[\theta_{0},\;\theta_{1},\;\ldots ,\;\theta_{i}\right]$ are the model parameters. The log-likelihood is widely used and can be computed as

(16) $$\log\;\ell \left(\theta \right)=\overset{L}{\underset{i=1}{\sum }}\log(({\left(g\left(\theta^{T}x^{i}\right)\right)}^{c\left(i\right)}\left(1-g\left(\theta^{T}x^{i}\right){)}^{1-c\left(i\right)}\right)\;$$

To avoid the overfitting, a regularisation term λ is added the log-likelihood function such that

(17) $$\log\;\ell \left(\theta \right)=\overset{L}{\underset{i=1}{\sum }}\log\left(P\left(c^{i}=c_{k}|x^{i};\theta \;\right)\right)-\frac{\lambda }{2}{∥\theta ∥}^{2}$$

The MLR is a linear supervised regression model that generalises the logistic regression (LR) where labels are binary, i.e., *c*^(*i*)^ ∈ {0,1} to multi-classification problems that have labels {1, …, *c*} where c is the number of classes. The LR and MLR have been used in machine fault diagnosis [52,53,54,55]. We briefly describe the simplified multinomial logistic regression model as follows: in multinomial logistic regression with multi-labels *c*^(*i*)^ ∈ {1, …, *c*} the aim is to estimate the probability *P*(*c* = *c*^(*i*)^ |*x*) for each value of *c*^(*i*)^ = 1 to *c*, such that

(18) $$h_{\theta }\left(x\right)=\left[\begin{array}{c} P\left(c=1|x;\theta \right)\\ P\left(c=2|x;\theta \right)\\ .\\ .\\ .\\ P(c=K|x;\theta ) \end{array}\right]=\frac{1}{\sum_{j=1}^{K}\exp\left(\theta^{\left(j\right)T}x\right)}\;\left[\begin{array}{c} e^{\theta^{\left(1\right)T}x}\\ e^{\theta^{\left(2\right)T}x}\\ .\\ .\\ .\\ e^{\theta^{\left(K\right)T}x} \end{array}\right]\;$$

where $\;\theta^{\left(1\right)}$,$\;\theta^{\left(2\right)}\;,\;\ldots ,\;\theta^{\left(K\right)}\;\in \;R^{n}$ are the parameters of the multinomial logistic regression model.

## 3. Experimental Study

The data used in this study were collected from a two-terminal simulation model of the MMC-HVDC transmission power system described in Section 2. Seven conditions of MMCs status have been recorded. These include one normal condition and six IGBT open-circuit fault conditions in lower and upper arms of the MMC containing, A-phase lower SMs, A-phase upper SMs, B-phase lower SMs, B-phase upper SMs, C-phase lower SMs, and C-phase upper SMs faults. A total of 100 examples of each condition were recorded; this gave a total of 700 (100 × 7) raw data files to work with. All the nine parameters—i.e., *I*_a_, *I*_b_, *I*_c_, *i_ap_*, *i_bp_*, *i_cp_*, *i_an_*, *i_bn_*, and *i_cn_*—were recorded to obtain 5001 time samples. The measurements of these nine parameters were concatenated to form a vector of samples that represent the current health condition of the MMCs. This gave a total of 45,009 (5001 × 9) samples dimension for each vector of health condition. First, experiments were conducted for testing data of sizes 15% to 50% and 20 trials for each experiment with 10-fold cross-validation.

By using PCA on the concatenated measurements and then ignoring all eigenvalues less than 10^−10^ of the largest eigenvalue, we obtain 604 principal components, retaining well over 99% of the total variance of the data. With these learned features, SVM-based on the ECOC algorithm and MLR algorithm are used for classification. Classification accuracies are obtained by averaging the results of twenty trials for each classifier and each experiment. Additionally, in each experiment, we have examined the effects of using a normalisation technique in our classification results for both SVM and MLR.

### 3.1. Results of SVM-Based ECOC without Data Normalisation

**Figure 6:**
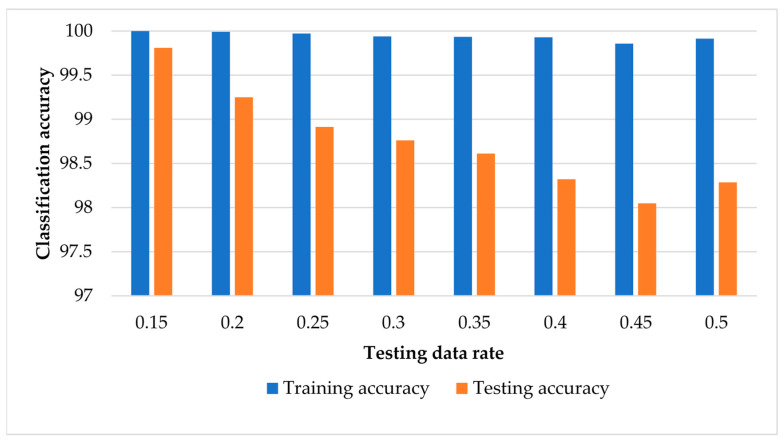
Classification results of training and testing data using SVM-based ECOC without data normalisation.

Figure 6 shows the classification results of training and testing data using our framework with an SVM-based ECOC classifier without data normalisation. On average, the testing classification results have high classification accuracies from 99.8% with 15% testing data to 98.0% with a 45% testing data rate. A possible explanation for these results might be that the increase in the training data might improve the accuracy of the trained classification model. Table 4 provides a sample confusion matrix of the classification results for each condition with testing data of 15% and 40%. As can be seen from Table 4, the recognition of the normal condition of the MMCs is 100% with both the 15% and 40% testing data. With 15% testing data, our method misclassified none of the testing examples of conditions 1, 3, 4, 5, and 7. For condition 2, our method misclassified 0.3% of the testing examples of condition 2 as condition 6 and 1% of the testing examples of condition 6 as condition 2. However, with 40% testing data, our method misclassified 1.2% of the testing examples of condition 2 as condition 4 (0.6%), condition 6 (0.4%), and condition 7 (0.2%) For condition 3, our proposed method misclassified 1.1% of the testing examples as condition 5 (0.3%) and condition 6 (0.7%). For condition 4, 0.9% of the testing examples were misclassified as condition 2 (0.4%) and condition 6 (0.5%). Results for condition 5 misclassified 3.4% of the testing examples as condition 2 (1.5%), condition 3 (0.8%), and condition 7 (1.1). Results for condition 6 showed that 1.8% of the testing examples were confused as condition 2, 0.4% of the testing examples were misclassified as condition 4, and 0.5% were misclassified as condition 7. Finally, 2.4% of the testing examples of condition 7 were misclassified as condition 2 (0.5%) and condition 5 (1.9%). 

### 3.2. Results of SVM-Based ECOC with Data Normalisation

**Figure 7:**
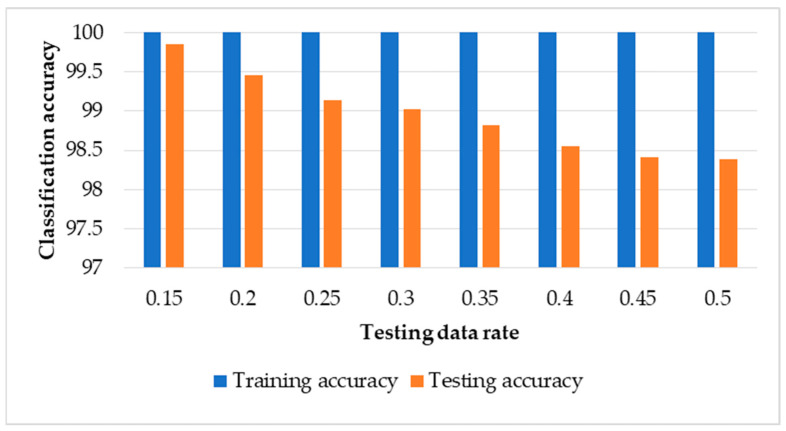
Classification results of training and testing data using SVM-based ECOC with data normalisation.

Figure 7 shows the classification results of training and testing data using our framework with the SVM classifier and data normalisation. Overall, the testing classification results have high classification accuracies where the minimum accuracy achieved is 98.4% with 50% testing data and maximum accuracy is 99.9% with 15% testing data. This indicates that there was a slight improvement in the classification accuracy of SVM-based ECOC with data normalization compared to the classification results without data normalization described above. Table 5 shows the sample confusion matrix of the classification results for each health condition with testing data of 15% and 40%. Additionally, the recognition of the normal healthy condition is 100% with both 15% and 40% of testing data. With 15% testing data, there are no misclassifications of conditions 1, 3, 4, 5, and 7. Results for condition 2 showed that 0.3% of the testing examples were confused as condition 6. While for condition 6, the results revealed that 0.7% of the testing examples were misclassified as condition 2. With 40% testing data, the recognition accuracy for condition 1—i.e., the normal condition—was 100%. For condition 2, 0.9% of the testing examples were misclassified as condition 4, 0.4% as condition 6, and 0.3% as condition 7. Results for condition 3 showed that 0.3% of the testing examples were misclassified as condition 5, and 0.6% as condition 6. For condition 4, 0.8% of the testing examples were confused as condition 2 and 0.1% as condition 6. The results for condition 5 showed that 1.5% of the testing examples were misclassified as condition 2, 0.8% were misclassified as condition 3, and 0.8% as condition 7. For condition 6, 1.5% were misclassified as condition 2, 0.4% as condition 3, and 0.6% as condition 7. Finally, for condition 7, 0.6% of the testing examples were misclassified as condition 2 and 0.5% as condition 5.

### 3.3. Results of MLR without Data Normalisation

**Figure 8:**
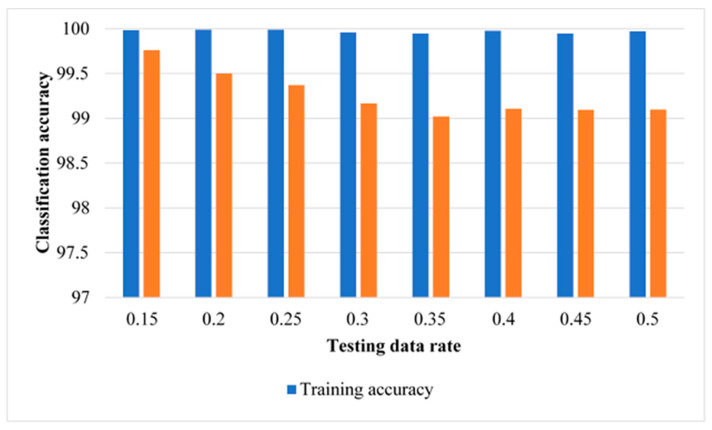
Classification results of training and testing data using MLR without data normalisation.

Figure 8 depicts the classification results of training and testing data using our framework with the MLR classifier without data normalisation. Figure 8 indicates that the testing results have classification accuracies above 99% for all the rates of testing data used in this study. In particular, classification results from our proposed method with MLR are 99.8% and 99.5% for testing data without normalisation of 15% and 20%, respectively. The least classification accuracy was achieved using testing data of 35% with 99%. Table 6 presents the sample confusion matrix of the classification results for each condition with testing data rates of 15% and 40%. With 15% testing data, there are no misclassifications of conditions 1, 3, 4, and 5—i.e., the recognition is 100%. Additionally, the results for condition 2 are misclassified 0.3% of the testing samples as condition 6. Moreover, results for condition 6 misclassified 1% of the testing samples as condition 2. Results concerning condition 7 were misclassified as condition 5. While with 40% of the testing data the recognition accuracy is 100% for conditions 1 and 5. However, results for condition 2 misclassified as condition 6 (0.4%) and as condition 7 (0.2%). For condition 3, 0.5% of the testing samples are misclassified as condition 5. For condition 4, 0.4% of the testing samples are misclassified as condition and 0.5% are misclassified as condition 6. Additionally, some testing examples of condition 6 are misclassified as condition 2 (2%), as condition 4 (0.4%), and as condition 7 (0.5%). Finally, results for condition 7 misclassified 0.5% of the testing examples as condition 2 and 0.9% as condition 5. Comparing Table 6 with Table 4 and Table 5 shows that overall classification accuracies are 99.8% (Table 4), 99.9% (Table 5), and 99.8% (Table 6) at 15% of testing data as well as 98.3% (Table 4), 98.6% (Table 5), and 99.1% (Table 6) at 40% of testing data.

### 3.4. Results of MLR with Data Normalisation

**Figure 9:**
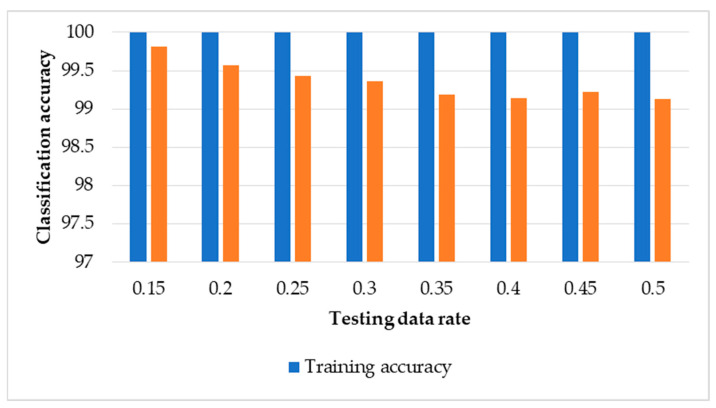
Classification results of training and testing data using MLR with data normalisation.

Figure 9 presents the classification results using our framework with the MLR classifier with data normalisation. Classification accuracies are above 99.0% for all the rates of testing data used in this study. For example, with normalised testing data of 15%, 20%, and 30%, our proposed method with MLR achieved 99.8%, 99.6%, and 99.4% respectively. Table 7 shows the sample confusion matrix of the classification results of MLR with data normalisation for each condition with testing data rates of 15% and 40%. Comparing this Table with Table 4, Table 5 and Table 6 shows that overall classification accuracies are 99.8% (Table 4), 99.9% (Table 5), 99.8% (Table 6), and 99.8% (Table 7) at 15% of testing data as well as 98.3% (Table 4), 98.6% (Table 5), 99.1% (Table 6), and 99.1% (Table 7) at 40% of testing data.

## 4. Comparisons of Results

In this section, the results achieved using our proposed framework with MLR and SVM-based ECOC are described. Classification results of the MMCs health conditions achieved using our proposed framework are compared with some recently published results.

### 4.1. Comparisons of Testing Classifications

**Figure 10:**
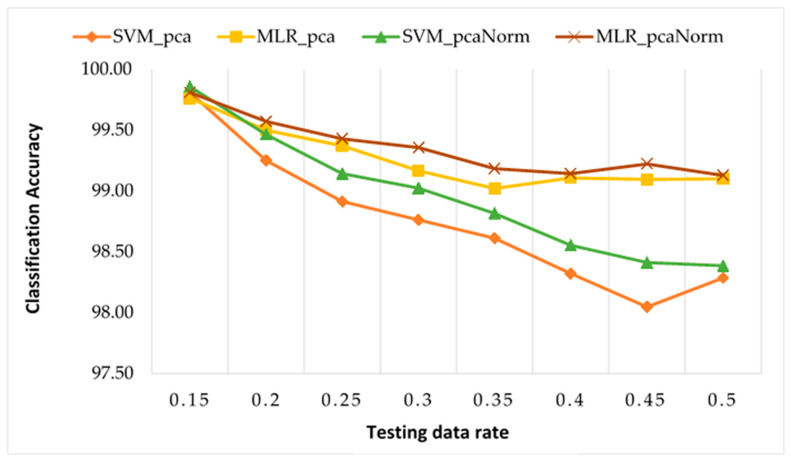
Comparisons of testing classification accuracies using our framework with SVM and MLR on normalised and unnormalised data.

Figure 10 provides a comparison of testing classification results achieved using our framework with SVM and MLR. From Figure 10, it is apparent that the classification accuracies of MMCs health condition using our framework with all four combinations are the same at 15% of the testing rate. Additionally, in each of MLR and SVM, results with normalised data are better than those with unnormalised data. For larger than 15% of the testing rate, MLR results are better than SVM results on both normalised and unnormalised data. Additionally, MLR testing takes significantly less than a 10th of the time of SVM.

### 4.2. Comparisons with Recently Published Results

Few studies have considered AI-based techniques for MMCs fault diagnosis. An adaptive one-dimensional convolutional neural network (CNN) based monitoring system was introduced in [27]; the raw voltage and current data were used directly as input to the CNN.

Another approach for MMC-HVDC DC fault detection and classification using CNN was proposed [56]. The wavelet decomposition and the wavelet logarithmic energy entropy (WLEE) were combined into the CNN to display their respective advantages based on the transient DC characteristics. In [57], a deep CNN (DCNN) algorithm and a combination of the voltage signals of the capacitors in all SMs were proposed for fault detection and identification.

Table 8 compares the recently published results of MMC fault diagnosis in [6,27,46,56,57] with ours. Although there are no direct comparisons due to different types of data used in [27,56,57], our proposed framework and the methods in [27,57] were compared in terms of the observed variables, number of measured parameters, number of health conditions used, fault detection accuracy, and testing time. The method used for MMC-HVDC DC fault detection in [56] achieved only 92.8% accuracy, while methods in [27,57], used for IGBT open-circuit fault diagnosis, obtain 98.9% and 98.2% accuracy respectively. All four procedures in our proposed framework offer better accuracies than all three of them and are significantly faster than the method in [27], even though we are using 100 examples of each condition compared to only five examples for each condition in [27].

Moreover, Table 8 shows further comparisons with recently published results in [6,36] using CNN, AE-DNN, LSTM, and a bidirectional LSTM (BiLSTM) with the same dataset at a 40% testing data rate. Two things are clear for each of the methods—(1) Classification results from our proposed framework, with SVM and MLR on both normalised and unnormalised data, outperform those achieved using CNN, AE-DNN, LSTM, and BiLSTM techniques; and (2) Our proposed framework with SVM and MLR is faster than the methods in [6,46]. For example, the reported results in [6] show that, with a 40% testing data rate, CNN achieved 97% classification accuracy and AE-DNN achieved 97.5% classification accuracy. In [38], with the same data and testing data rate, LSTM achieved 97% classification accuracy and BiLSTM achieved 97% classification accuracy. While our proposed framework with the same data (unnormalised) used in [6,46] achieved 98.3% and 99.1% classification accuracy using SVM and MLR respectively. Moreover, our proposed framework with both MLR and SVM is much faster than CNN, AE-based DNN, LSTM, and BiLSTM that were studied in [6,46]. As can be seen in Table 8, the results illustrate that our proposed framework with MLR requires only 1% of the time of the CNN, 0.27% of the time of the AE-DNN, 0.6% of the time of the LSTM, and only 0.3% of the time of the BiLSTM. Additionally, our proposed framework with ECOC-based SVM requires 26.5% of the time of the CNN, 7% of the time of the AE-DNN, 8% of the time of the LSTM, and only 4% of the time of the BiLSTM.

## 5. Conclusions

This study has developed and evaluated an intelligent diagnosis framework for automatically recognizing MMCs faults in HVDC transmissions. An original framework based on feature extraction and classification algorithms with a combination of the AC-side three-phase current and the upper and lower bridge’ currents of each of the three phases of the MMCs has been proposed. PCA has been used to reduce the data dimension by selecting significantly fewer components possessing most of the variance of the original measurements of the current signals. With these selected components, a classifier (SVM or MLR) has been used to classify the health conditions of the MMC. A two-terminal simulation model of the MMC high-voltage direct current (HVDC) transmission power system using PSCAD/EMTDC software was used to validate the proposed framework for MMCs faults diagnosis in HVDC transmissions. In each experiment, we have examined the effects of using a normalisation technique for both SVM and MLR. Classification results from our proposed framework with MLR are better than those with SVM on both normalised and unnormalised data. Comparisons with recently published results based on machine learning indicate that our framework with the MLR classifier is faster by an order of magnitude, and our classification accuracies with SVM and MLR are better than the others. For example, the results illustrate that our proposed framework with MLR requires only 1% of the time of the CNN, 0.27% of the time of the AE-DNN, 0.6% of the time of the LSTM, and 0.3% of the time of the BiLSTM, and yet our classification results are better. The reduced computational time of our proposed method is achieved as a result of using a minimum number of only current sensors as well as using the PCA method to select fewer features to train the classification algorithm—i.e., SVM and MLRC algorithms—and to classify the MMC-HVDC’s health conditions using the trained classification models.

## Figures and Tables

**Table 1:** Summary of different techniques that have been used in different studies of IGBT open-circuit fault diagnosis.

Ref.	Approach Used	Detection Threshold Parameters	Localisation Threshold Parameters
[24]	Fault detection: The comparison between the observed ${\hat{i}}_{p}$ using Sliding Mode Observer (SMO) and measured the current of the upper arm *i_p_*. Fault localisation The comparison between the observed and measured lower arm current, ${\hat{i}}_{p}$ and *i_p_*, and the capacitor voltages, ${\hat{V}}_{ci}$ and *V_ci_* of the assumed faulty SM.	Threshold parameters: *I_th_*, and 700 time-steps (2 µs per step) Usage: If $\left|\;{\hat{i}}_{p}-i_{p}\right|\geq I_{th}$ and it lasts for 700 time-steps (2 µs per step) then an open-circuit fault has occurred.	Threshold parameters: *I_th_*, *V_thi_*, and 100 ms. Usage: If $\left|\;{\hat{i}}_{p}-i_{p}\right|<I_{th}$ and $\left|\;{\hat{V}}_{ci}-V_{ci}\right|<V_{thi}$ and it lasts for 100 ms, then the SM is faulty.
[42]	Fault detection: The detection is achieved using a state observer to estimate the ideal circulating current ${\hat{i}}_{c}$ and the output current ${\hat{i}}_{o}$ using the state models of the MMC and the variables already available from the main control system. Fault localisation The comparison between the SM capacitor voltage *V_ci_* and a threshold value *U_th_*.	Threshold parameters: *I^th^*, and Δ*T*_1_. Usage: If $\left|\;{\hat{i}}_{c}-i_{c}\right|>I_{th}\;$ and it lasts for Δ*T*_1_, then an open-circuit fault has occurred.	Threshold parameters: *U^th^*, and Δ*T*_2_. Usage: If $V_{ci}\geq U_{th}$ and it lasts for Δ*T*_2_, then this SM is located to be faulty.
[44]	Fault detection: The comparison between measured inner difference current *i_diff_* and the estimated inner difference current ${\hat{i}}_{diff}$ through Kalman Filter (KF). Fault localisation Each capacitor voltage (*V_c,p,N_*) of the upper and lower arms of the targeted phase, i.e., the phase with a detected fault, is compared with the minimum capacitor voltage value (*V*_*c*(*t*),*min*_) for a given time instant.	Threshold parameters: Δ*i_diff_* and Δ*t_i_*. Usage: If $\left|{\hat{i}}_{diff}-i_{diff}\right|>\Delta i_{diff}$ and it lasts for a period of Δ*t_i_*, then a fault is assumed in this phase of the MMC.	Threshold parameters: Δ*V_c_* and Δ*t_v_*. Usage: If $V_{c,\;p,\;N}-V_{c\left(t\right),min}>\Delta V_{c}\;\Delta V_{c}$ and it lasts for a period of Δ*t_v_*, then it indicates this SM is faulty.
[45]	Fault detection: The comparison between the measured current of the lower arm current *i_N_* and observed arm current ${\hat{i}}_{N}$, which is based on Sliding Mode Observer (SMO), and one of the capacitor voltages, ${\hat{V}}_{c}$ and *V_c_*. Fault localisation The comparison between the observed and measured lower arm current, ${\hat{i}}_{N}$ and *i_N_*, and the capacitor voltages, ${\hat{V}}_{ci}$ and *V_ci_* of the assumed faulty SM.	Threshold parameters: *I_th_* and *V_th_*. Usage: If $\left|\;{\hat{i}}_{N}-i_{N}\right|\geq I_{th}$ and $\left|\;{\hat{V}}_{c}-V_{c}\right|\geq ,\;V_{th}$ and it lasts for 500 μs (50 time-steps, 10 μs per step), then an open-circuit fault has occurred.	Threshold parameters: *I_th_* and *V_thi_*. Usage: If $\left|\;{\hat{i}}_{N}-i_{N}\right|<I_{th}$, and $\left|\;{\hat{V}}_{ci}-V_{ci}\right|<V_{thi}$ and it lasts for 80 ms, then the SM is faulty.

**Table 2:** Parameters of MMC [6].

Parameters	Value
Number of SMs per arm	9
SM capacitor	3000 μF
Arm inductance	0.05 ohm
AC frequency	50 Hz

**Table 3:** MMC health conditions [6].

Faulty Bridge	Label Value
Normal	1
A-phase lower SMs	2
A-phase upper SMs	3
B-phase lower SMs	4
B-phase upper SMs	5
C-phase lower SMs	6
C-phase upper SMs	7

**Table 4:** Sample confusion matrix of the classification results of SVM-based ECOC without data normalization.

** Testing Data = 15% **							
	**Normal**	**A-Phase Lower SMs**	**A-Phase Upper SMs**	**B-Phase Lower SMs**	**B-Phase Upper SMs**	**C-Phase Lower SMs**	**C-Phase Upper SMs**
Normal	100	0	0	0	0	0	0
A-Phase Lower SMs	0	99.7	0	0	0	1	0
A-Phase Upper SMs	0	0	100	0	0	0	0
B-Phase Lower SMs	0	0	0	100	0	0	0
B-Phase Upper SMs	0	0	0	0	100	0	0
C-Phase Lower SMs	0	0.3	0	0	0	99.0	0
C-Phase Upper SMs	0	0	0	0	0	0	100
** Testing Data = 40% **							
	**Normal**	**A-Phase Lower SMs**	**A-Phase Upper SMs**	**B-Phase Lower SMs**	**B-Phase Upper SMs**	**C-Phase Lower SMs**	**C-Phase Upper SMs**
Normal	100	0	0	0	0	0	0
A-Phase Lower SMs	0	98.8	0	0.4	1.5	1.8	0.5
A-Phase Upper SMs	0	0	98.8	0	0.8	0	0
B-Phase Lower SMs	0	0.6	0	99.1	0	0.4	0
B-Phase Upper SMs	0	0	0.5	0	96.6	0	1.9
C-Phase Lower SMs	0	0.4	0.7	0.5	0	97.4	0
C-Phase Upper SMs	0	0.2	0	0	1.1	0.5	97.6

**Table 5:** Sample confusion matrix of the classification results of SVM-based ECOC with data normalisation.

** Testing Data = 15% **							
	**Normal**	**A-Phase Lower SMs**	**A-Phase Upper SMs**	**B-Phase Lower SMs**	**B-Phase Upper SMs**	**C-Phase Lower SMs**	**C-Phase Upper SMs**
Normal	100	0	0	0	0	0	0
A-Phase Lower SMs	0	99.7	0	0	0	0.7	0
A-Phase Upper SMs	0	0	100	0	0	0	0
B-Phase Lower SMs	0	0	0	100	0	0	0
B-Phase Upper SMs	0	0	0	0	100	0	0
C-Phase Lower SMs	0	0.3	0	0	0	99.3	0
C-Phase Upper SMs	0	0	0	0	0	0	100
** Testing Data = 40% **							
	**Normal**	**A-Phase Lower SMs**	**A-Phase Upper SMs**	**B-Phase Lower SMs**	**B-Phase Upper SMs**	**C-Phase Lower SMs**	**C-Phase Upper SMs**
Normal	100	0	0	0	0	0	0
A-Phase Lower SMs	0	98.5	0	0.8	1.5	1.5	0.6
A-Phase Upper SMs	0	0	98.9	0	0.8	0	0
B-Phase Lower SMs	0	0.9	0	99.1	0	0.4	0
B-Phase Upper SMs	0	0	0.5	0	97.0	0	0.5
C-Phase Lower SMs	0	0.4	0.6	0.1	0	97.5	0
C-Phase Upper SMs	0	0.3	0	0	0.8	0.6	98.9

**Table 6:** Sample confusion matrix of the classification results of MLR-based without data normalisation.

** Testing Data = 15% **							
	**Normal**	**A-Phase Lower SMs**	**A-Phase Upper SMs**	**B-Phase Lower SMs**	**B-Phase Upper SMs**	**C-Phase Lower SMs**	**C-Phase Upper SMs**
Normal	100	0	0	0	0	0	0
A-Phase Lower SMs	0	99.7	0	0	0	1	0
A-Phase Upper SMs	0	0	100	0	0	0	0
B-Phase Lower SMs	0	0	0	100	0	0	0
B-Phase Upper SMs	0	0	0	0	100	0	0.3
C-Phase Lower SMs	0	0.3	0	0	0	99.0	0
C-Phase Upper SMs	0	0	0	0	0	0	99.7
** Testing Data = 40% **							
	**Normal**	**A-Phase Lower SMs**	**A-Phase Upper SMs**	**B-Phase Lower SMs**	**B-Phase Upper SMs**	**C-Phase Lower SMs**	**C-Phase Upper SMs**
Normal	100	0	0	0	0	0	0
A-Phase Lower SMs	0	99.4	0	0.4	0	2	0.5
A-Phase Upper SMs	0	0	99.5	0	0	0	0
B-Phase Lower SMs	0	0	0	99.1	0	0.4	0
B-Phase Upper SMs	0	0	0.5	0	100	0	0.9
C-Phase Lower SMs	0	0.4	0	0.5	0	97.1	0
C-Phase Upper SMs	0	0.2	0	0	0	0.5	98.6

**Table 7:** Sample confusion matrix of the classification results of MLR-based with data normalisation.

**Testing Data = 15%**							
	**Normal**	**A-Phase Lower SMs**	**A-Phase Upper SMs**	**B-Phase Lower SMs**	**B-Phase Upper SMs**	**C-Phase Lower SMs**	**C-Phase Upper SMs**
Normal	100	0	0	0	0	0	0
A-Phase Lower SMs	0	99.7	0	0	0	1	0
A-Phase Upper SMs	0	0	100	0	0	0	0
B-Phase Lower SMs	0	0	0	100	0	0	0
B-Phase Upper SMs	0	0	0	0	100	0	0
C-Phase Lower SMs	0	0.3	0	0	0	99.0	0
C-Phase Upper SMs	0	0	0	0	0	0	100
**Testing Data = 40%**							
	**Normal**	**A-Phase Lower SMs**	**A-Phase Upper SMs**	**B-Phase Lower SMs**	**B-Phase Upper SMs**	**C-Phase Lower SMs**	**C-Phase Upper SMs**
Normal	100	0	0	0	0	0	0
A-Phase Lower SMs	0	99.4	0	0.4	0	2	0.5
A-Phase Upper SMs	0	0	99.5	0	0.2	0	0
B-Phase Lower SMs	0	0	0	99.1	0	0.4	0
B-Phase Upper SMs	0	0	0.5	0	99.8	0	0.4
C-Phase Lower SMs	0	0.4	0	0.5	0	97.1	0
C-Phase Upper SMs	0	0.2	0	0	0	0.5	99.1

**Table 8:** Our results with 10-fold cross validation compared with some recently published results.

Ref.	Type of Measurement	No. of Measured Parameters	Classification Accuracy	Testing Time
[27]	Capacitor voltage Circulating current	5000 × 7 5 × 9	98.9%	80 ms
[56]	DC current	--	92.8%	-
[57]	Capacitor’s voltages in all SMs	800 × 72	98.2%	--
[6]	Current signals CNN AE-DNN	5001 × 9 40% testing rate	97.0% 97.5%	400 ms 1500 ms
[46]	Current signals LSTM BiLSTM	5001 × 9 40% testing rate	97.4% 97.0%	1290 ms 2630 ms
Proposed framework at 15% testing rate using PCA in all cases	Current signals and their phases	5001 × 9 100 × 7		
	SVM, no norm		99.8%	62 ms
	SVM, with norm		99.9%	59 ms
	MLR, no norm		99.8%	4 ms
	MLR, with norm		99.8%	4 ms
at 40% testing rate using PCA in all cases	SVM, no norm		98.3%	106 ms
	SVM, with norm		98.6%	96 ms
	MLR, no norm		99.1%	8 ms
	MLR, with norm		99.2%	7 ms

## Data Availability

The data presented in this study may be available on request from the first author, Hosameldin O. A. Ahmed. The data are not publicly available due to privacy reasons.
